# Persistent delirium is associated with cerebrospinal fluid markers of neuronal injury

**DOI:** 10.1093/braincomms/fcae319

**Published:** 2024-09-18

**Authors:** Alex Tsui, Benjamin Johnstone, Amanda Heslegrave, Henrik Zetterberg, Leiv Otto Watne, Bjørn Erik Neerland, Maria Krogseth, Colm Cunningham, Alasdair MacLullich, Graciela Muniz Terrera, Daniel Davis, Gideon Caplan

**Affiliations:** Department of Population Science and Experimental Medicine, MRC Unit for Lifelong Health and Ageing at University College London (UCL), 1-19 Torrington Place, London WC1E 7HB, UK; St Pancras Rehabilitation Unit, Central and North West London NHS Foundation Trust, London NW1 0PE, UK; St Pancras Rehabilitation Unit, Central and North West London NHS Foundation Trust, London NW1 0PE, UK; UK Dementia Research Institute at University College London (UCL), London W1T 7NF, UK; UK Dementia Research Institute at University College London (UCL), London W1T 7NF, UK; Department of Neurochemical Pathophysiology and Diagnostics, University of Gothenburg, 405 30 Gothenburg, Sweden; Oslo Delirium Research Group, Department of Geriatric Medicine, Oslo University Hospital, 0318 Oslo, Norway; Oslo Delirium Research Group, Department of Geriatric Medicine, Oslo University Hospital, 0318 Oslo, Norway; Oslo Delirium Research Group, Department of Geriatric Medicine, Oslo University Hospital, 0318 Oslo, Norway; Institute of Neurology, Trinity College Dublin, Dublin, D02 PX31, Ireland; Queen’s Medical Research Institute, University of Edinburgh, Edinburgh EH16 4TJ, UK; Usher Institute Centre for Population Health Sciences, University of Edinburgh, Edinburgh EH16 4UX, UK; Heritage College of Osteopathic Medicine, Ohio University, Athens, Ohio 45701, USA; Department of Population Science and Experimental Medicine, MRC Unit for Lifelong Health and Ageing at University College London (UCL), 1-19 Torrington Place, London WC1E 7HB, UK; St Pancras Rehabilitation Unit, Central and North West London NHS Foundation Trust, London NW1 0PE, UK; Department of Geriatric Medicine, Prince of Wales Hospital, Sydney, 2031, Australia; University of New South Wales, Sydney, 2052, Australia

**Keywords:** delirium, dementia, CSF biomarkers, neurofilament light chain, glial fibrillary acidic protein

## Abstract

Delirium is associated with the risk of future long-term cognitive impairment, but the degree to which markers of neuronal injury may be distinct or shared with dementia has yet to be comprehensively described. We investigated CSF biomarkers of dementia, astrocytosis and neuronal damage in a clinical cohort with persistent delirium, comparing them with an outpatient memory clinic sample. Our aim was to determine if different patterns of biomarker changes could implicate specific mechanisms for delirium-related neuronal injury over and above that attributable to comorbid dementia. We recruited 35 participants from the Prince of Wales Hospital, Sydney, Australia. We included inpatients with delirium persisting for at least 5 days (*n* = 15, 10 with underlying dementia) and participants from outpatient memory clinics (*n* = 20, 17 with dementia). CSF assays were as follows: amyloid-β_42_, amyloid-β_40_, phosphorylated tau181, neurofilament light chain and glial fibrillary acidic protein. We used propensity score matching to estimate effect sizes for each standardized CSF biomarker separately for persistent delirium (irrespective of underlying dementia) and dementia (irrespective of superimposed delirium). Compared with individuals without delirium, persistent delirium was associated with elevated glial fibrillary acidic protein (normalized coefficient per transformed standard deviation, *β* = 0.85; 95% confidence interval: 0.03–1.68) and neurofilament light chain (*β* = 1.1; 95% confidence interval: 0.5–1.6), but not phosphorylated tau181. Compared with individuals without dementia, glial fibrillary acidic protein, neurofilament light chain and phosphorylated tau181 were all increased to expected levels in dementia cases, with the former two biomarkers at levels comparable to those seen in persistent delirium [glial fibrillary acidic protein (*β* = 1.54; 95% confidence interval: 1.05–2.0) and neurofilament light chain (*β* = 0.65; 95% confidence interval: 0.24–1.1)]. Persistent delirium was linked with changes in CSF biomarkers not necessarily attributable to dementia. These findings support the potential that delirium is associated with direct neuronal injury independent of dementia pathophysiology. Whether this neuronal injury involves astrocyte dysfunction or direct axonal damage are both possibilities. Future work examining acute brain injury in delirium is needed.

## Teaser

Persistent delirium poses risks for long-term cognitive impairment, but how neuronal injury markers relate to dementia remains unclear. Tsui *et al*. studied CSF biomarkers in medical admissions with persistent delirium and dementia, finding elevated glial fibrillary acidic protein and neurofilament light chain, suggesting direct neuronal injury beyond dementia-related changes.

## Graphical Abstract

**Graphical Abstract:**
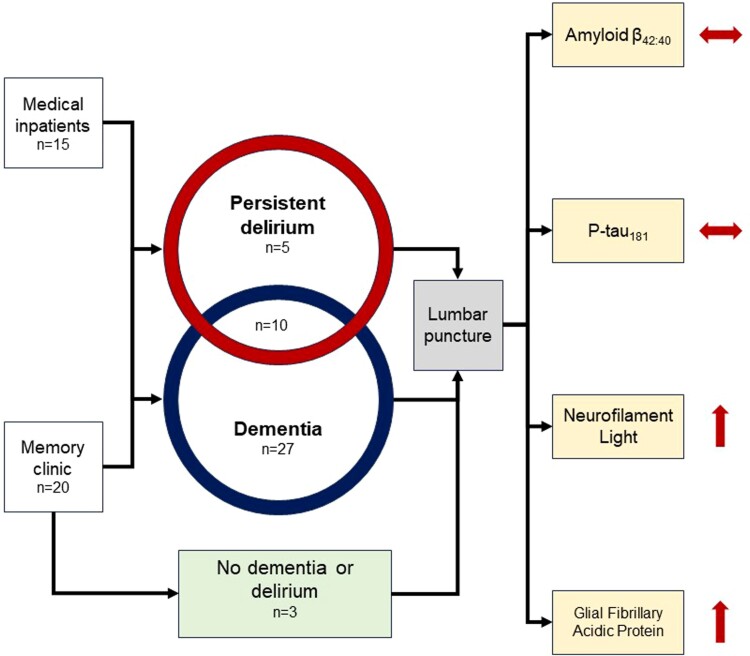

## Introduction

Delirium is a neuropsychiatric syndrome characterized by acute inattention and global cognitive impairment, which tends to fluctuate, precipitated by a physiological insult.^1,2^ Although classically considered to recover over hours and days after resolution of the acute precipitant, studies show that in a substantial proportion of patients, delirium persists, with up to 16% of episodes still evident after 12 months.^3^ The association of delirium with subsequent cognitive decline is well established. Further work has shown that the strength of this relationship is related to both the severity and duration of delirium episodes, further supporting the importance of the delirium–dementia link and the need for more research on the underpinning mechanisms.^4-6^

There have been some recent advances in understanding delirium pathophysiology.^2^ However, the degree to which it might be distinct or shared with underlying dementia is unclear.^7^ Dementia is the leading risk factor for delirium, and CSF biomarkers identified in Alzheimer’s disease and other dementias could plausibly contribute to delirium-related adverse outcomes. Amyloid-beta (Aβ) precursor protein is cleaved into Aβ_42_ and Aβ_40_ isoforms; a reduced Aβ_42:40_ ratio suggests Alzheimer’s pathology.^8^ Tau protein phosphorylated at threonine181 (P-tau181) forms neurofibrillary tangles pathognomonic for Alzheimer’s disease. Neurofilament light chain (NfL) is a neuroaxonal skeletal polypeptide elevated in several stable neurodegenerative diseases, indicating neuronal cell damage and eventual death.^9,10^ Glial fibrillary acidic protein (GFAP), an astrocyte cytoskeletal filament protein marker of astrogliosis and astrocyte degeneration, is elevated in neurodegeneration and correlated to Alzheimer’s disease severity.^11^

In clinical delirium studies, various biomarkers have implicated neuroinflammatory or neuroendocrine abnormalities, with more recent work identifying impaired glucose utilization, and a role for the kynurenine pathway.^12-15^ Plasma NfL levels rise in correlation with perioperative delirium severity and predict more prolonged delirium and worse outcomes in critically ill patients.^16,17^ Although over 90% of hospitalized delirium presents in emergency or medical contexts,^18^ CSF samples have generally been obtained from surgical cohorts, in whom the subarachnoid space is often accessed for anaesthetic purposes.^19^ Few studies have studied CSF neurodegeneration and neuronal injury biomarkers in inpatient and outpatient settings and even fewer have been in acutely unwell medical patients.

We set out to investigate neuropathological biomarkers for delirium in a clinical cohort of acutely unwell medical patients with persistent delirium, defined as delirium that had not resolved within 5 days. Our overall aim was to determine if different patterns of biomarker levels could imply specific mechanisms for delirium-related neuronal injury over and above that attributable to comorbid dementia. Specifically, we hypothesized that patients with dementia would be associated with Aβ_42:40_ ratio, GFAP, NfL and P-tau181 as per prior studies in stable populations. We also hypothesized that persistent delirium would be associated with both altered dementia biomarkers and also biomarkers of more acute change, namely, GFAP and NfL, and persistent delirium, implicating acute axonal neuronal damage and astrocyte dysfunction.

## Materials and methods

We followed the REDEEMS guidelines (checklist in Supplementary Table 1).^20,21^

### Recruitment

Participants were recruited between June 2007 and June 2018 at the Prince of Wales Hospital, a tertiary hospital in the South Eastern Sydney Area Health Service, Australia. Eligible patients were aged ≥65 admitted to a geriatric medicine inpatient ward and with persistent delirium (duration ≥ 5 days) despite treatment of identified reversible causes, diagnosed according to DSM 5 criteria by a specialist geriatrician, and in whom it was clinically appropriate to rule out central nervous system aetiologies. Patients with hearing loss were engaged with using hearing aids and amplifiers as required. Interpreters were used for patients for whom English was not their first language. Patients undergoing surgery or treated with palliative intent were excluded. Any procedural contraindication, lack of clinical indication to lumbar puncture and inability to obtain patient or proxy consent were exclusion criteria.

Our comparison group consisted of patients referred to the outpatient memory clinic for the investigation of a possible dementia diagnosis. They were concurrently assessed for and excluded if demonstrating a diagnosis consistent with incident delirium.

### Clinical ascertainment

We collected baseline medical, socio-demographic and functional data on the day of the lumbar puncture: years of completed formal schooling, previous occupation, past medical diagnoses (including continence and falls history), prescribed medications, Informant Questionnaire on Cognitive Decline in Elderly (IQCODE), previous mini-mental state examinations (MMSE) administered for clinical purposes, instrumental activities of daily living (IADLs), Barthel ADL Index, Geriatric Depression Score (GDS), mobility aids, Charlson Comorbidity Index, alcohol and smoking history, accommodation type and any formal care arrangements.^22-27^ We documented baseline function related to participants’ status 2 weeks before the assessment date. We used the acute component of APACHE II score (FiO2, temperature, mean arterial pressure, heart rate, respiratory rate, sodium, potassium, creatinine, haematocrit, white cell count and Glasgow Coma Scale) to quantify the severity of acute illness.

Delirium was evaluated from the day of admission, up to 10 times during inpatient stay, using the Confusion Assessment Method (CAM) and Delirium Index (DI), which gave a delirium diagnosis for each assessment. We used a consensus panel to determine dementia status (AT, DD, GC, all geriatricians experienced in dementia diagnosis), using all data from clinical assessments. These included IQCODE and GDS scores, any impairment of activities of daily living and previously documented dementia diagnoses. All participants recruited from the memory clinic were assessed for delirium as part of their clinical evaluation; none did in this setting.

### CSF collection and analyses

CSF samples were collected after day 5 of delirium by lumbar puncture on the ward or under X-ray guidance, using local anaesthetic without sedation. We used polypropylene tubes, gently inverted three to four times, and centrifuged (2000 g, 10 min, +4°C) to remove cells and debris. CSF was transferred to a clean polypropylene tube, and 50, 100 and 250 μL aliquots were dispensed into labelled cryovials and stored at −80°C until required. Samples were sent under dry ice conditions by air freight to London, UK. All CSF analyses were performed by the Biomarker Laboratory of the UK Dementia Research Institute at UCL by operators blinded to the clinical diagnosis, an accredited laboratory with a standardized operating protocol.^28^

### Statistical analyses

We investigated differences in those with persistent delirium versus those without. Simultaneously, we compared those with dementia to those without. We followed this approach throughout the analysis, i.e. persistent delirium versus no delirium (irrespective of underlying dementia) and dementia versus no dementia (irrespective of superimposed persistent delirium). Differences in clinical characteristics were established using Fisher’s exact test (categorical data) and Wilcoxon rank sum tests (continuous data). For each CSF biomarker, heteroskedasticity was quantified first by visual inspection of histograms of corrected replicate concentration. We chose the best transformations guided by the *gladder* function on Stata. Each transformed biomarker was centred and mean standardized.

We used propensity score matching to assess the associations for persistent delirium or dementia with each collected biomarker. Statistical analyses of small sample sizes are challenging, and a conservative approach is necessary to limit the chance of Type I errors. Several techniques have evolved that appear robust to addressing imbalances in covariates for which sample sizes would be too small for standard regression. Propensity score matching is a valid approach with small samples, provided that the component regression models are not overfitted (details below); simulation studies support this method by comparing decreasing samples from *n* = 1000 to *n* = 40 without appreciable increases in Type I error rate.^29^

**Figure 1:**
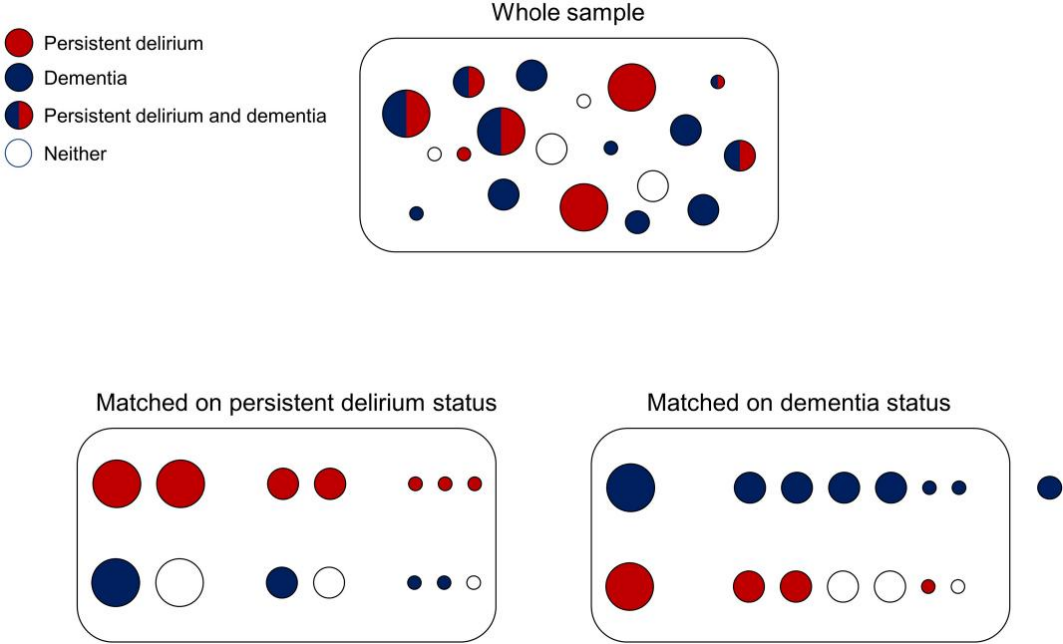
**Schematic for propensity score matching**. Two models to assess differences in biomarker concentrations (outcomes) based on individuals with (i) *persistent delirium* or (ii) *dementia*. Circles of different sizes represent differences in matching variables [age, sex, illness acuity (APACHE II score)]; one dementia case could not be matched (sitting outside the box). From the whole sample, those with persistent delirium can be matched to non-delirium cases, irrespective of dementia and vice versa.

Propensity score matching uses data from the whole sample to identify distinct profiles associated with persistent delirium or dementia (Fig. 1), allowing relationships to be identified not otherwise apparent by examining raw scores. The probability of having persistent delirium (irrespective of underlying dementia) or dementia (irrespective of superimposed delirium) is estimated based on logistic regression, conditioned on covariates affecting both the outcome and exposure.^30^ This allowed us to derive a propensity score for each participant, i.e. the degree to which an individual, given their age, sex and other variables such as acute illness, would be likely to have persistent delirium or dementia. If propensity scores for either condition are sufficiently similar for matching, then we could estimate if biomarker levels differed between cases and controls. We used the *teffects psmatch* function in Stata (tmodel: logit, stat: ate), with a calliper (the maximum difference between propensity scores required to determine a match) set at 0.25.^30^ As recommended, any data points without a match were removed from subsequent analyses for that exposure. We used an iterative process to optimize propensity score balance and overlap by trialling inclusion and exclusion of covariates to ensure standardized differences of all covariates to be less than 0.25.

The final covariates for inclusion of the persistent delirium model were age, sex, APACHE score (acute component) and dementia; the final covariates for the dementia model were age, sex, APACHE score (acute component) and persistent delirium, thereby fitting the most parsimonious model allowed by the sample size. One participant did not have an APACHE score recorded; here, we imputed it from another participant matched within 1 year of age, identical sex, persistent delirium and dementia status, Barthel Index and IADL scores. We reported the effect sizes for both models as standardized coefficients for each biomarker, with significance testing of maximum likelihood estimates derived from Wald *χ*^2^ tests.

Stata 17.0 (StataCorp, Texas) was used for all statistical analyses.

### Ethics

Ethics approval was obtained from the Human Research Ethics Committee of the South-Eastern Sydney Health Area (Eastern Section), the University of New South Wales Ethics Committee and the NSW Guardianship Tribunal.

## Results

**Figure 2:**
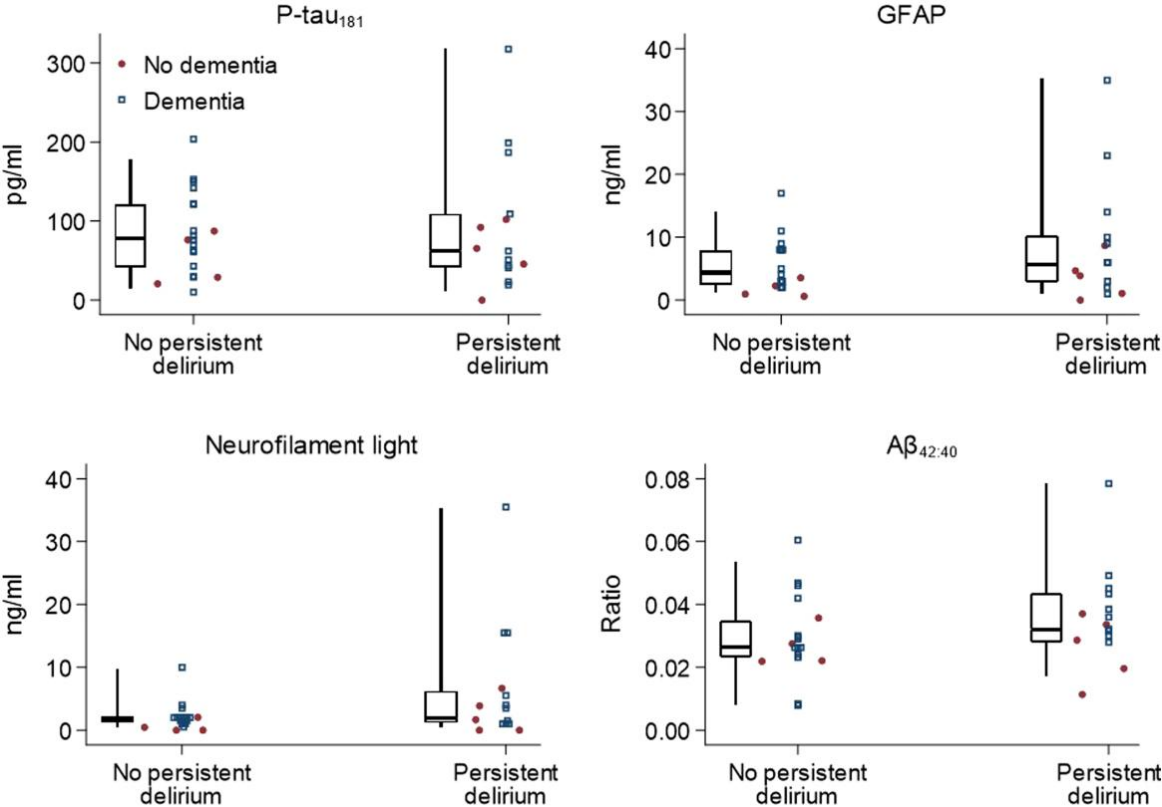
**Dot and box plot of CSF biomarkers by persistent delirium**. P-tau181, phosphorylated tau 181; GFAP, glial fibrillary acidic protein; NfL, neurofilament light chain; Aβ, amyloid-beta. Dot plots show the same results by dementia status, with jitter added to non-dementia cases for clarity. One individual with dementia and neurofilament light chain level = 100 ng/mL *not shown*.

Of 35 participants, 15 were inpatients with persistent delirium, and 20 were recruited from memory clinic. The median age was 84% and 43% were men (Table 1). We determined dementia in 27 participants (10 with persistent delirium and 17 from the memory clinic sample). Other than higher Delirium Index and APACHE scores, patients with persistent delirium were more likely to have lower Barthel scores (Table 1). Those with dementia were more likely to have IADL impairments and receive formal care (Table 1). For raw CSF biomarker levels, NfL and GFAP, P-tau181 and Aβ_42:40_ ratio had acceptable intraplate coefficients of variation (Supplementary Table 2) and were not statistically different in those with persistent delirium. However, NfL levels were skewed to higher levels in persistent delirium (Table 1; Fig. 2). In dementia, all biomarkers were elevated in the expected directions, except for Aβ_42:40_ ratio (*P* = 0.22) (Table 1; Fig. 2).

**Table 1:** Summary of baseline characteristics and CSF biomarkers

	Persistent delirium (*n* = 15)	No persistent delirium (*n* = 20)	*P*
Age	85 (82–88)	83 (81–86)	0.44
Men	5 (33%)	10 (50%)	0.32
Charlson Comorbidity Index	6 (5–6.5)	6 (5–6)	0.97
Geriatric Depression Scale	7 (4.3–8)	6 (4–7.5)	0.28
Delirium Index	9 (7.5–14)	3 (2–3)	<0.01
APACHE II	39 (30–42)	35 (31–49)	0.01
Barthel Index	17 (13–20)	20 (20–23)	<0.01
IQCODE	3.9 (3.2–4.1)	4.1 (3.7–5.0)	0.05
IADL impairments	2 (0–4)	9 (7–12)	0.22
Alcohol history	1 (7%)	6 (30%)	0.10
Smoking history	1 (7%)	2 (10%)	0.76
Mobility aid use	13 (87%)	5 (25%)	<0.01
Formal care	10 (66%)	11 (55%)	0.52
P-tau181 (pg/mL)	62.5 (42.4–108.9)	78.7 (41.5–121.1)	0.66
GFAP (ng/mL)	5.72 (2.89–10.2)	4.34 (2.44–7.91)	0.59
NfL (ng/mL)	1.84 (1.20–6.23)	1.95 (1.25–2.58)	0.52
Aβ_42_ (pg/mL)	124.2 (74.8–186.6)	128.5 (96.9–179.0)	0.97
Aβ_40_ (pg/mL)	3462 (2483–4454)	4388 (3213–5198)	0.07
Aβ_42:40_ ratio	0.032 (0.028–0.043)	0.026 (0.023–0.034)	0.09

	Dementia (*n* = 27)	No dementia (*n* = 8)	*P*
Age	84 (82–87)	82 (73–87)	0.18
Men	14 (52%)	1 (13%)	0.05
Charlson Comorbidity Index	6 (5–6)	4.5 (3.5–7)	0.10
Geriatric Depression Scale	6 (4–7)	8 (2.5–10.5)	0.26
Delirium Index	4 (3–9)	4.5 (1.75–8.25)	0.96
APACHE II	31 (27–39)	37 (31.5–28.25)	0.68
Barthel Index	19 (15–20)	20 (19.5–20)	0.15
IQCODE	4.1 (3.7–4.5)	3.3 (3.3–3.3)	0.05
IADL impairments	4 (3–7)	10 (7.75–12)	<0.01
Alcohol history	5 (19%)	1 (13%)	0.67
Smoking history	3 (11%)	0 (0%)	0.63
Mobility aid use	11 (41%)	2 (25%)	0.38
Formal care	19 (70%)	2 (25%)	0.02
P-tau181 (pg/mL)	76.4 (43.6–142)	50.7 (19.7–74.3)	0.05
GFAP (ng/mL)	6.32 (3.06–9.24)	3.18 (1.82–4.51)	0.03
NfL (ng/mL)	2.12 (1.42–4.14)	1.19 (0.77–1.55)	0.02
Aβ_42_ (pg/mL)	131.3 (97.1–186.7)	106.9 (51.9–129.0)	0.24
Aβ_40_ (pg/mL)	4142 (2723–5001)	3481 (2502–4630)	0.53
Aβ_42:40_ ratio	0.030 (0.026–0.043)	0.025 (0.023–0.035)	0.22

Numbers accompanied by interquartile range or %.

APACHE II, Acute Physiology and Chronic Health Evaluation II (acute component only); IQCODE, Informant Questionnaire on Cognitive Decline in the Elderly; IADL, Instrumental Activities of Daily Living; P-tau181, phosphorylated tau-181; GFAP, glial fibrillary acid protein; Aβ, amyloid-beta.

We derived propensity scores for 35 and 34 patients for *persistent delirium* and *dementia* status, respectively. One patient was excluded from the dementia model as no match could be found within pre-determined calliper limits, that is, standardized differences were below 0.25 for all covariates in the final persistent delirium model and for all covariates except age in the dementia model (Supplementary Fig. 1A and C). Overall, propensity score overlaps were satisfactory (Supplementary Fig. 1B and D).

**Figure 3:**
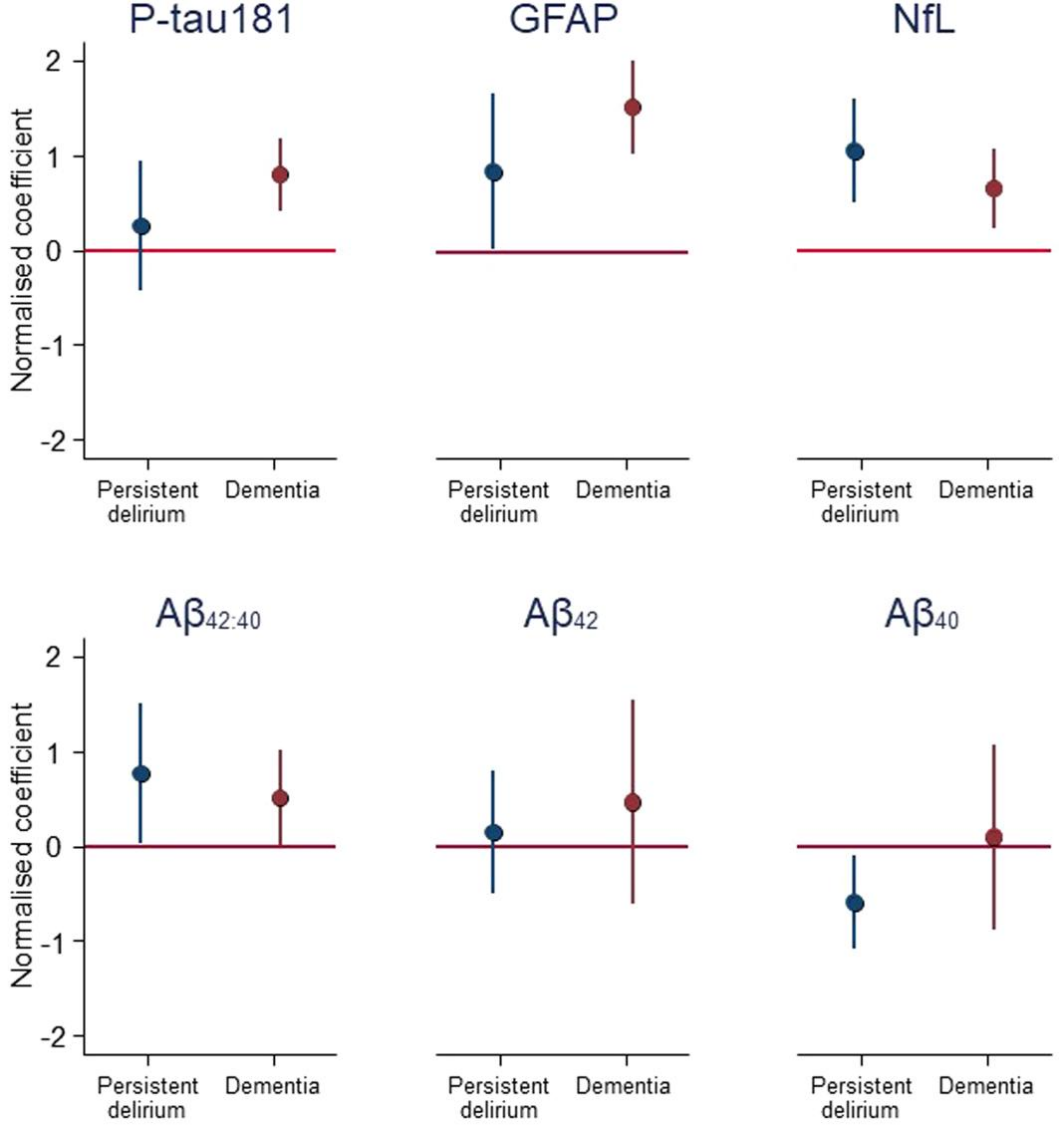
**Comparison of normalized coefficients for effects of persistent delirium (compared with no delirium) and dementia (compared with no dementia), per SD of transformed CSF biomarkers**. P-tau181, phosphorylated tau 181; GFAP, glial fibrillary acidic protein; NfL, neurofilament light chain; Aβ, amyloid-beta. Normalized coefficients on *y*-axis represent SDs of log-transformed biomarker concentrations from adjusted linear regression with significance testing of maximum likelihood estimates derived from Wald *χ*^2^ tests (raw data points given in Fig. 2).

Persistent delirium was associated with higher GFAP [normalized coefficient per transformed standard deviation (SD), *β* = 0.85; 95% confidence interval (CI): 0.03–1.68] and NfL (*β* = 1.1; 95% CI: 0.51–1.6), as well as lower CSF Aβ_40_ (though not Aβ_42_) concentrations (*β* = −0.59; 95% CI: −1.1 to −0.10) (Table 2; Fig. 3). Dementia was associated with higher GFAP (*β* = 1.54; 95% CI: 1.05–2.0), NfL (*β* = 0.65; 95% CI: 0.24–1.1) and P-tau181 (*β* = 0.80; 95% CI: 0.42–1.2).

**Table 2:** Normalized coefficients for effect estimates of persistent delirium and dementia per SD of each transformed CSF markers

	Normalized coefficient	95% CI		*P*
Persistent delirium model (*n* = 35)				
P-tau181	0.26	−0.43	0.94	0.46
GFAP	0.85	0.03	1.68	0.04
NfL	1.05	0.51	1.59	<0.01
Aβ_42:40_ ratio	0.77	0.02	1.51	0.04
Aβ_42_	0.15	−0.50	0.79	0.66
Aβ_40_	−0.59	−1.09	−0.10	0.02
Dementia model (*n* = 34)				
P-tau181	0.80	0.42	1.18	<0.01
GFAP	1.54	1.05	2.04	<0.01
NfL	0.65	0.24	1.06	<0.01
Aβ_42:40_ ratio	0.51	0.02	1.01	0.04
Aβ_42_	0.47	−0.62	1.55	0.40
Aβ_40_	0.10	−0.88	1.07	0.84

One participant in dementia model could not be matched. All biomarkers except Aβ_42:40_ ratio are log transformed.

## Discussion

The findings suggest persistent delirium is associated with elevated CSF NfL and GFAP but not P-tau181. In persistent delirium, GFAP and NfL were increased to levels comparable to concentrations seen in dementia. Moreover, persistent delirium was associated with reduced Aβ_40_, but not Aβ_42_, where both were lower in dementia. Taken together, these results demonstrate that persistent delirium may be linked with changes in CSF biomarkers not necessarily attributable to dementia, strengthening the possibility that delirium is associated with direct neuronal injury independent of pathophysiology typical of Alzheimer’s disease and other dementias.

Our analyses are subject to certain limitations. First, the small sample size precluded adjustment using standard linear regression because of the risk of overfitting.^31^ Nonetheless, we wanted to address differences in baseline demographics between the persistent delirium and dementia groups to understand the true underlying relationships, and propensity score matching is increasingly used in observational studies.^32^ It offers a compromise rooted in causal inference approaches that are valid in small sample sizes^29^ yet can account for appropriate confounders. Using this method, we achieved balanced overlap within acceptable standardized differences to estimate and compare relative effect sizes. The current binary constructs for persistent delirium and dementia do not capture severity or duration (e.g. cumulative delirium burden). Observational associations may be subject to residual confounding. We could not differentiate specific delirium aetiologies or dementia subtypes; while certain dementia substrates or delirium precipitants may have contributed variably to biomarkers levels, our data nonetheless reflects clinical reality, in which a broader spectrum of multiple pathophysiologies likely coexist in unwell older populations. Similarly, acute illness is not a uniform entity fully captured by APACHE II score. Some medical illnesses may have led to a direct elevation in brain injury markers (e.g. GFAP, NfL and potentially P-tau-181). For example, syncope could be hypothesized to elevate GFAP acutely as a result of brain hypoxia or as a result of a fall with minor head trauma. Lastly, selection biases arise when only considering delirium persisting for at least 5 days (and investigated by lumbar puncture) that may not apply to delirium in general. Similarly, patients referred to a memory clinic subsequently determined to have no dementia will have differences with the general population. However, this comparator might lead to underestimating the differences in persistent delirium. Nonetheless, these are the first analyses to show that persistent delirium is directly associated with pathophysiological markers of astrocyte and neuronal injury in a medical cohort where CSF samples are rarely available.

Our findings are consistent with previous studies implicating long-term cognitive impairment after delirium as distinct from dementia uncomplicated by delirium. Population studies with neuropathology samples have suggested delirium is associated with incident dementia and long-term cognitive decline independently of Alzheimer’s pathology.^33^ Moreover, other longitudinal cohorts and meta-analyses have shown dose-dependent long-term cognitive decline in association with delirium.^4-6^ Our results add to this understanding by identifying potential mechanisms contemporaneous with persistent delirium.

Elevated CSF NfL suggests neuroaxonal damage contributes to the pathophysiology of persistent delirium. Increased plasma and CSF NfL have been shown in delirium patients in elective surgery, emergency orthopaedic surgery and critical care settings.^17,34-36^ In elective surgical patients, no preoperative differences were demonstrated in plasma NfL, but postoperative NfL increased dose dependently with delirium severity.^17^ This temporal correlation implicates NfL in delirium pathophysiology, as opposed to changes being attributable to prior subclinical pathology. In a mixed cohort of elective and emergency surgical patients, activation of the kynurenine pathway and elevated quinolinic acid was associated with delirium, high NfL and mortality.^15^ Increased NfL indicating neuroaxonal damage fits with our previous report of reduced CSF ApoE, which has an important role in neuroaxonal membrane repair.^13^ Our finding that CSF P-tau181 was not increased in persistent delirium supports the idea that pre-existing subclinical dementia was less likely to be a contributing factor. A marker of astrocyte dysfunction, GFAP appears sensitive but not specific for neurodegenerative conditions.^37-40^ This may contribute to persistent delirium insofar as it reflects brain vulnerability to metabolic stress and other insults. While we did not demonstrate any change in Aβ_42:40_ ratio in persistent delirium, we did find a specific decrease in CSF Aβ_40_. The diagnostic value of lower Aβ_42:40_ ratio for Alzheimer’s pathology is based on Aβ_42_ being a constituent of neuritic plaques. Aβ_40_ concentrations are relatively stable and predominate in the periphery, including being the principal isoform deposited in systemic vasculature. It may be that acute illness and delirium perturb CSF amyloid-β_40_ levels, perhaps through changes in intracerebral or peripheral production, peripheral binding, access to the CNS or breakdown by intracerebral macrophage recruitment.^41,42^

Future research requires replication in larger prospective longitudinal serial samples associated with long-term outcomes. Practically, serial biomarker ascertainment throughout a delirium episode would need to be blood based, and GFAP and NfL are attractive in this respect. These need to be at the same time as more granular clinical assessments, accounting for different time courses, severities, aetiologies and baseline cognitive impairments. By incorporating baseline and extended prospective sample collections, serial biomarker measurements can highlight chain interactions between acute and premorbid illness states, including distinguishing primed or superadded effects. Ultimately, we would like to understand how persistent delirium might evolve from an abnormal, though at least partially reversible state, to one more typical of a chronic and irreversible dementia. Prognostic biomarkers could act as molecular stratifiers to identify patients most suitable for inclusion in future therapeutic trials. Lastly, investigating how acute illness impacts amyloid-β metabolism might show delirium as relevant for understanding amyloid clearance therapies.

In conclusion, better understanding of persistent delirium pathophysiology opens new possibilities for identifying specific mechanisms for cognitive impairment distinct from pathophysiology typical of Alzheimer’s disease. Whether these mechanisms involve astrocyte dysfunction or direct axonal damage are both possibilities.

## Supplementary Material

fcae319_Supplementary_Data

## Data Availability

Source codes are published on the MRC Unit for Lifelong Health and Ageing at UCL GitHub. Clinical and biomarker data are available for sharing on request (g.caplan@unsw.edu.au).
