# Supplementary material for: Persistent delirium is associated with cerebrospinal fluid markers of neuronal injury
Source: Brain Commun. 2024 Sep 18;6(5):fcae319. doi: 10.1093/braincomms/fcae319 (PMC11443447; doi:10.1093/braincomms/fcae319)
Supplement: fcae319_Supplementary_Data [file fcae319_supplementary_data.pdf]

Supplementary methods

Amyloid beta 42 and 40 (AB42, 40)

CSF sAPPα, sAPPβ (cat. no. K15120E), NfL, GFAP (in-house assays as previously described in detail,[1,2] were measured by ELISA. P-tau (cat. no. 230350), amyloid β 1–40 (Aβ40; cat. no. 231524), and 1–42 (Aβ42; cat. no. 230336) were analysed by an architectural platform (xMAP Multiplex; Luminex Corporation) and a kit (INNO-BIA AlzBio3; Fujirebio). The Aβ42/Aβ40 ratio was calculated and used as a marker of amyloid accumulation.[3]

In brief, Innogenetics kit reagents included well-characterised capture monoclonal antibodies specific for $A\beta_{1-42}$(4D7A3), t-tau(AT120), and p-tau_{181p} (AT270), each chemically bonded to unique sets of color-coded beads, and analyte-specific detector antibodies (HT7, 3D6). Calibration curves were produced for each biomarker using aqueous buffered solutions that contained the combination of three biomarkers at concentrations ranging from 56 to 1,948pg/ml for recombinant tau, 27 to 1,574pg/ml for synthetic $A\beta_{1-42}$ peptide, and 8 to 230pg/ml for a tau synthetic peptide phosphorylated at the threonine 181 position (ie, the p-tau_{181p} standard).

Tau markers (p-tau)

The levels of CSF p-$tau_{(181P)}$ was determined using a sandwich ELISA (INNOtest® hTAU-Ag p-$Tau_{(181P)}$; Fujirebio Europe N.V., Gent, Belgium) constructed to measure both normal tau and phosphorylated tau. Briefly, for the hTAU Ag assay, tau protein is captured from CSF samples by a monoclonal anti-tau antibody (AT120) bound to a microtiter plate. Captured tau is detected with two biotinylated tau-specific monoclonal antibodies (HT7 and BT2). Similarly, for the t-tau assay, p-$tau_{(181P)}$ is captured from CSF samples by anti-tau antibody HT7 bound onto a microtiter plate. Captured p-$tau_{(181P)}$ is detected with a biotinylated monoclonal anti-p-tau antibody (AT270). In both assays, peroxidase-labelled streptavidin and tetramethylbenzidine (TMB) substrate are also added. Peroxidase-catalysed hydrolysis produces a colorimetric signal. Sample concentrations are extrapolated from a standard curve, fitted using a 4-parameter logistic algorithm. Intra-assay CVs were less than 20%.

Neurofilament light (NFL)

NFL was measured using the commercially available NF-Light ELISA, according to the manufacturer's instructions (UmanDiagnostics, Umeå, Sweden). Briefly, samples were diluted 1:2 with sample diluent and added in duplicate to microplate wells coated with a monoclonal capture antibody specific for NFL. Samples were incubated with a biotinylated NFL-specific monoclonal detection antibody. The detection complex was completed with the addition of horseradish peroxidase-labelled streptavidin and TMB substrate. Peroxidase-catalysed hydrolysis produces a colorimetric signal. Sample concentrations were extrapolated from a standard curve, fitted using a

4-parameter logistic algorithm. Intra-assay CVs were less than 10%. Samples were run on two different days by different operators; the inter-assay CV was below 16%.

Glial fibrillary acidic protein (GFAP)

CSF GFAP levels were quantified for all cohorts on the Simoa HD-X (Quanterix) using the commercial single-plex assay (No. 102336).

Supplementary Figure 1

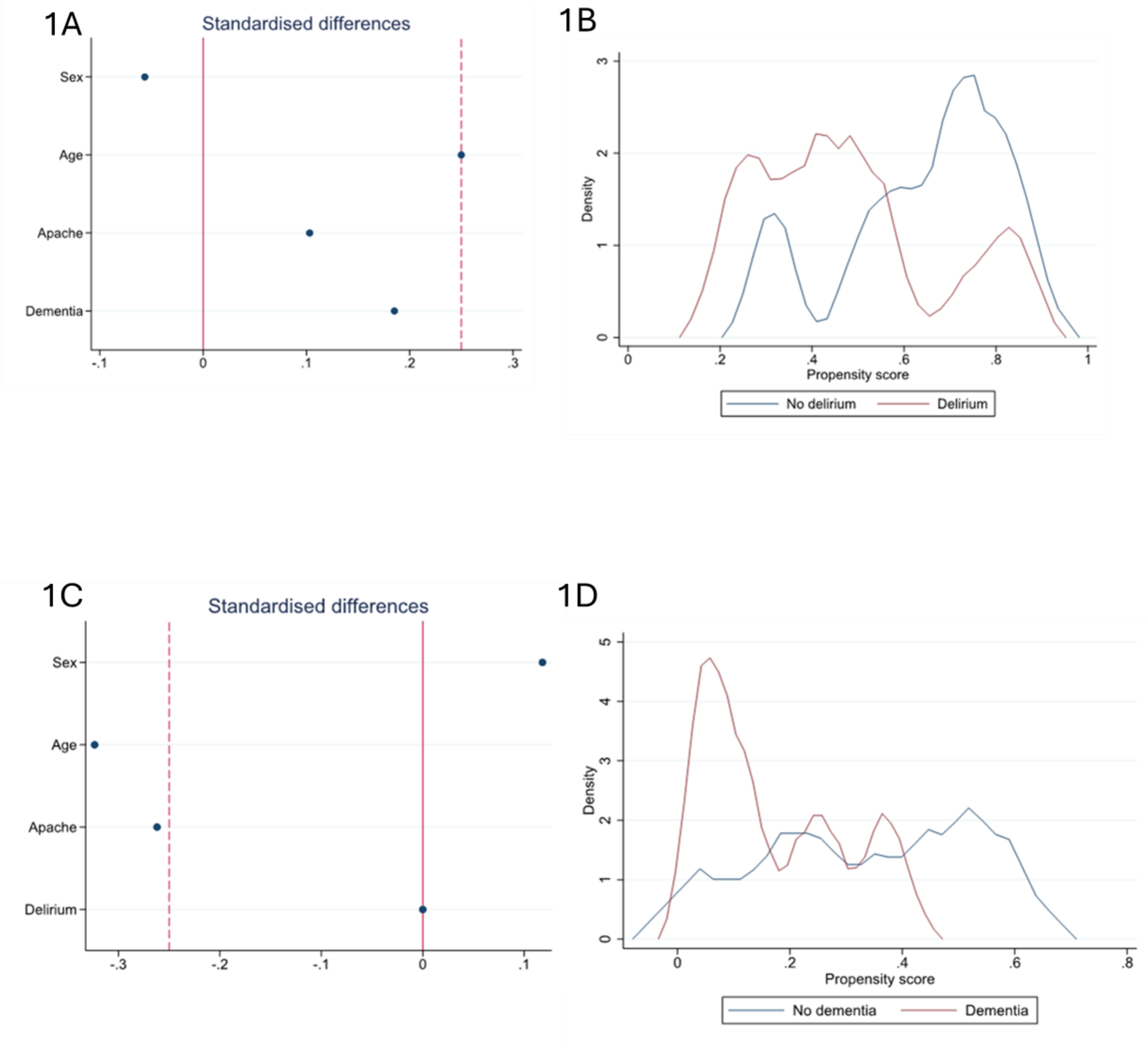

1A: standardised differences for propensity score matching for delirium model. **1B**: propensity score overlap for delirium and no delirium as exposures. **1C:** standardised differences for propensity score matching for dementia model. **1D:** propensity score overlap for dementia and no dementia as exposures.

Supplementary Table 1: REDEEMS guidelines

Item number	REDEEMS items
**1**	**Study rationale**
a	State the biomarker under study (including nature of the specimen)<br><br>Cerebrospinal fluid neurofilament light, phosphorylated tau 181, glial fibrillary acidic protein, amyloid beta 40, amyloid beta 42<br><br>p3 para 2
b	Describe the biological hypothesis(/es) tested*<br><br>CSF biomarkers in patients with delirium may quantitively differ from patients with dementia alone<br><br>p3 para 2
**2**	**Ascertainment of delirium**
a	Describe the training and/or credentials of personnel who ascertained delirium cases<br><br>Delirium was ascertained by a consultant geriatrician<br><br>p5 para 2
b	Specify the delirium tool and/or diagnostic process that was used to ascertain cases<br><br>Confusion assessment method (CAM) and delirium index (DI)<br><br>p5 para 2
c	Describe frequency, timing and duration of delirium assessment<br><br>Daily from day of admission, at random times of working day, up to ten times during inpatient day.<br><br>p5 para 2
**3**	**Outcome measures**
a	Define and justify all clinical endpoint(s) and their measures (including relationship to delirium where relevant)<br><br>CSF biomarkers<br><br>p5 para 3
**4**	**Assay procedure**

Item number	REDEEMS items
a	Specify the assay method used with a detailed protocol that includes reagents/kits<br><br>p5 para 3<br>Supplementary material
b	Describe the methods of preservation, storage and processing of the biological sample<br><br>p5 para 3<br>Supplementary material
c	Describe the assay validation method for repeatability and robustness including the sensitivity limits of the assay<br><br>Supplementary material
d	Specify the inter- and intra- assay coefficients of variation<br><br>Supplementary material
e	Specify the method of blinding biomarker results<br><br>Biomarker assays were performed by an independent research lab blinded to delirium/ dementia status of each patient<br><br>p5 para 3
**5**	**Timing of collection of the biological sample**
a	Precisely describe the time of collection of the biological sample in relation to delirium (onset, presence, resolution)<br><br>CSF collected after day 5 of ongoing persisting delirium without resolution<br><br>p5 para 3
b	Provide a rationale for the timing of the sample collection based on the clinical scenario, the hypothesis being tested, and/or the study design<br><br>Five days offer sufficient ascertainment of delirium with regards to its inherent fluctuations, while including sufficient “dosage” to maximise biomarker signal<br><br>p5 para 2
**6**	**Confounding variables**
a	State the confounding variables assessed and whether or not they were specified *a priori*

Item number	REDEEMS items
	Age, sex, Apache score (acute component) and persistent delirium/ dementia diagnosis<br><br>p7 para 2
b	Clearly define and provide justification for the confounding variables (including the relationship to delirium where relevant)<br><br>All have hypothesised effects on delirium/ dementia as an outcome<br><br>p7 para 2
**7**	**Sample size**
a	Describe how sample size was determined and provide a rationale<br><br>The total sample size was opportunistically determined by clinical need for further investigation of alternative aetiologies of persistent delirium and patient/ next of kin consent<br><br>p5 para 3
**8**	**Statistical analysis**
a	Account for clinical and biomarker missing data in the analysis plan based on the design of the study<br><br>There were no missing biomarkers or delirium data. One missing APACHE score was imputed to nearest similar patient with comparable other demographics and measures of acute illness<br><br>p7 para 2
b	State how confounding variables were accounted for in the analysis<br><br>Appropriate confounders were included into the propensity score construction of each designated exposure<br><br>p7 para 2
**9**	**Univariate and multivariable analysis**
a	Report the estimated effect size or the p values with their Confidence Intervals (CI)<br><br>NA – no multivariate/ univariate models were used.
b	Specify whether the biomarker was dichotomised using a cut-point and/or threshold

Item number	REDEEMS items
	Biomarker was a continuous outcome measure<br><br>p6 para 3
c	Specify the number of included participants and reasons for attrition or missing data<br><br>Delirium model – all (35) patients included<br>Dementia model – 34 patients included, one excluded due to being outside caliper threshold for matching of propensity scores<br><br>p7 para 2
d	Describe how model assumptions were verified (multivariable)<br><br>NA – no multivariate/ univariate models were used.

* If the study is not testing a specific hypothesis, authors should state that it is undertaking an un-biased or exploratory approach

Supplementary Table 2: Intraplate coefficients of variations

	Intra-Plate %CV		
**Plex**	**average**	**stdev**	**%CV**
AB40	163.3686117	3.985435137	2.439535414
AB42	6.995413781	0.263530674	3.767192087
GFAP	155.7382131	6.176725396	3.966094943
NFL	29.89151973	1.342539092	4.491371145
pTau-181	3.807900714	0.250452889	6.577190643

Supplementary references

1. Gaetani L, Höglund K, Parnetti L, et al. A new enzyme-linked immunosorbent assay for neurofilament light in cerebrospinal fluid: analytical validation and clinical evaluation. *Alzheimers Res Ther*. 2018;10(1):8. doi:10.1186/s13195-018-0339-1

2. Rosengren LE, Ahlsén G, Belfrage M, Gillberg C, Haglid KG, Hamberger A. A sensitive ELISA for glial fibrillary acidic protein: application in CSF of children. *J Neurosci Methods*. 1992;44(2-3):113-119. doi:10.1016/0165-0270(92)90004-w

3. Hansson O, Lehmann S, Otto M, Zetterberg H, Lewczuk P. Advantages and disadvantages of the use of the CSF Amyloid β (Aβ) 42/40 ratio in the diagnosis of Alzheimer’s Disease. *Alzheimers Res Ther*. 2019;11(1):34. doi:10.1186/s13195-019-0485-0
